# Functional brain network organization supports cognitive function in patients with Alzheimer’s Disease‐related pathological burden and brain atrophy

**DOI:** 10.1002/alz.088708

**Published:** 2025-01-09

**Authors:** Ziwei Zhang, Micaela Chan, Ezra Winter‐Nelson, Gagan S Wig

**Affiliations:** ^1^ University of Texas at Dallas, Richardson, TX USA; ^2^ University of Texas Southwestern Medical Center, Dallas, TX USA

## Abstract

**Background:**

Alzheimer’s disease (AD) is defined by the accumulation of Aβ plaques and tau neurofibrillary tangles. The consequences of these pathologies include neurodegeneration and cognitive dysfunction. However, the process by which AD pathologies leads to cognitive impairment remains unclear. Some individuals can harbor pathological burden yet be cognitively unimpaired; these observations have led to ideas about cognitive reserve (Stern, Lancet, 2012).

Dominant models of AD biomarkers do not include direct measures of brain function. To address this gap, we tested whether resting‐state brain system segregation explains discrepancies between AD‐related pathology, structural atrophy, and cognitive function. System segregation changes across the adult lifespan, is stratified in relation to life course exposures, and relates to cognitive function (Wig, Trends in Cognitive Sciences, 2017).

**Method:**

Participants were recruited through the Alzheimer’s Disease Neuroimaging Initiative. Cognitive impairment was quantified using clinical dementia rating (CDR). The participant group included 184 healthy (CDR=0) adults, 101 very mild dementia (CDR=0.5), 24 mild dementia (CDR=1) and 2 moderate dementia (CDR=2) patients (age range: 55–96 years).

Functional brain networks were generated using resting‐state fMRI time series. Network edges were defined as correlations between time series of each pair of nodes. Brain system segregation was calculated to quantify functional brain network organization for each participant (Chan et al., PNAS, 2014).

**Result:**

Multiple linear regression analyses were used to test whether Aβ and tau relate to brain system segregation, with age, gender, education, and head motion included as covariates. Neither Aβ nor tau burden was associated with brain system segregation (Aβ: p=0.45; tau: p=0.55).

Among individuals exhibiting positive pathological burden (either Aβ, tau, or both) based on cut‐off values, individuals with higher system segregation were less cognitively impaired (Aβ+: p=0.007; tau+: p=0.021; Aβ and tau+: p=0.03).

Finally, system segregation explained unique variance with respect to CDR status over and above Aβ, tau, and cortical thickness. Brain system segregation was negatively related to CDR status (p=0.003), while accounting for these other factors.

**Conclusion:**

Functional brain network organization supports observations of cognitive reserve, and explains unique variance with respect to AD‐related cognitive impairment independent of AD‐related pathology and neurodegeneration.

